# Design of a novel analogue peptide with potent antibiofilm activities against *Staphylococcus aureus* based upon a sapecin B-derived peptide

**DOI:** 10.1038/s41598-024-52721-0

**Published:** 2024-01-26

**Authors:** Nasim Akhash, Ahmad Farajzadeh Sheikh, Zahra Farshadzadeh

**Affiliations:** 1 Health Research Institute, Infectious and Tropical Diseases Research Center, Ahvaz Jundishapur University of Medical Sciences, Ahvaz, Iran; 2 Department of Microbiology, Faculty of Medicine, Ahvaz Jundishapur University of Medical Sciences, Ahvaz, Iran

**Keywords:** Drug discovery, Microbiology

## Abstract

Nowadays, antimicrobial peptides are promising to confront the existing global crisis of antibiotic resistance. Here, a novel analogue peptide (mKLK) was designed based upon a D-form amidated sapecin B-derived peptide (KLK) by replacing two lysine residues with two tryptophan and one leucine by lysine, and inserting one alanine. The mKLK displayed superior amphipathic helixes in which the most of hydrophobic residues are confined to one face of the helix and had a higher hydrophobic moment compared with KLK. The mKLK retained its antibacterial activity and structure in human serum, suggesting its stability to proteolytic degradation. The values of MIC and MBC for mKLK were equal to those of KLK against clinical strains of methicillin-resistant *Staphylococcus aureus* (MRSA) and methicillin-susceptible *Staphylococcus aureus* (MSSA). However, mKLK showed more capability of in vitro inhibiting, eradicating, and dispersing MRSA and MSSA biofilms compared with KLK. Furthermore, a remarkable inhibitory activity of mKLK against MRSA and MSSA biofilms was seen in the murine model of catheter-associated biofilm infection. Results of this study show that mKLK not only exhibits antibacterial activity and serum stability but also a potent biofilm inhibitory activity at sub-MIC concentrations, confirming its potential therapeutic advantage for preventing biofilm-associated MRSA and MSSA infections.

## Introduction

*Staphylococcus aureus* is an important human pathogen causing a wide variety of clinical infections such as bacteremia and pneumonia^1,2^. Successful treatment of *S. aureus* remains a significant challenge due to the emergence of multi-drug resistant strains including Methicillin-Resistant ones (MRSA)^2^. In addition to antibiotic resistance, biofilm formation capacity plays a significant role in the pathogenicity of *S. aureus*^3^. Generally, biofilms are composed of a structured community of microbial cells (bacteria and/or fungi) embedded surrounded in a complicated polymeric matrix, which consists of exopolysaccharides, nucleic acids (eDNA and eRNA), proteins, lipids, and other biomolecules. Biofilms represent a protective mechanism by which bacteria survive in harsh environments and become more resistant to clearance by immune responses and to treatment with antibiotics^3–5^. There is a pressing need to discover and develop novel antibiofilm agents, as conventional antibiotics are unable to eradicate approximately 5–10% of bacterial biofilms^6^.

Today, antimicrobial peptides (AMPs) have attracted great attention as a potent alternative to traditional antibiotics^7–9^. Because of the importance of the cell membrane in pathogen viability, membrane-targeted antimicrobial agents such as AMPs can greatly hamper bacterial viability. AMPs have been considered a promising category of new antimicrobials, as they exhibit broad-spectrum antimicrobial activity and are less affected by mechanisms underlying bacteria antibiotic resistance^10^. Although extensive resistance to many classes of conventional antibiotics has emerged, the highly conserved and essential nature of the bacterial membrane would indicate a decreased potential for bacteria to be resistant to AMPs^11,12^. However, the susceptibility of AMPs to proteases restricts their pharmaceutical applications. To combat this challenge, instead of L-amino acids AMPs can be synthesized from D-amino acids, resulting in D-form peptides with more resistance to degradation by proteases^13,14^. The C-terminal amidation of AMP is another strategy by which the susceptibility of AMP to proteolytic degradation is reduced. Moreover, the C-terminal amidation enhances the antibacterial potency compared with the original peptide with a free carboxylic acid at the C-terminus due to the increment of the helicity and cationic charge by removing the negative charge of the carboxyl group, consequently enhancing peptide binding to negatively charged bacterial membranes^15–17^.

To improve the antibacterial potency, stability, and selectivity of cationic AMPs, the substitution or incorporation of hydrophobic amino acid residues into their sequences has been substantially explored^18^. The unique physicochemical properties of the amino acid tryptophan allow it to be involved in several interactions such as π-interactions, hydrogen bonds, and hydrophobic contacts. The extensive π–electron system of the aromatic indole side chain confers the ability of tryptophan to participate in π-interactions with various membrane components, thereby promoting increased peptide–membrane interactions^19,20^. All these interactions make tryptophan a key element for the antibacterial action of membrane-targeted peptides. The peptides possessing strong antibiofilm activity exhibit a higher abundance of hydrophobic amino acids such as tryptophan^21^. Antibiofilm peptides containing tryptophan residues could disrupt quorum sensing and effectively inhibit biofilm formation^22^. Molecular analysis of *S. aureus* quorum-sensing revealed an efficient interaction between the quorum-sensing protein, *AgrA*, and the tryptophan’s indole ring. Moreover, tryptophan significantly reduces the expression of the quorum-sensing gene (*agrA*)^23^. The position of tryptophan in the peptide sequence can influence antimicrobial potency. The peptides with an accumulation of tryptophans at the N-terminus of the peptide sequences seemed to be more efficient as antibacterial agents^21–24^. The arrangement of Trp residue in the N‐terminus significantly increased the antibacterial activity of the peptides, but the arrangement of this amino acid in the C‐terminus reduced their biocompatibility^25^.

The KLK peptide (KLKLLLLLKLK-NH_2_) is a C-terminal amidated D-form synthetic AMP derived from sapecin B, a peptide of Sarcophaga peregrine^17^. The KLK peptide was found to exhibit antimicrobial activities against gram-negative and gram-positive bacteria, and fungi. Numerous studies have demonstrated the potent antimicrobial activity of KLK against *S. aureus*^17,26,27^. To our knowledge, no study was conducted to investigate the antibiofilm properties of KLK. The present study aimed to design a peptide based upon KLK to increase its antibiofilm activity against *S. aureus* strains through lysine to tryptophan substitution in N‐terminus.

## Results

### Peptide design

**Figure 1:**
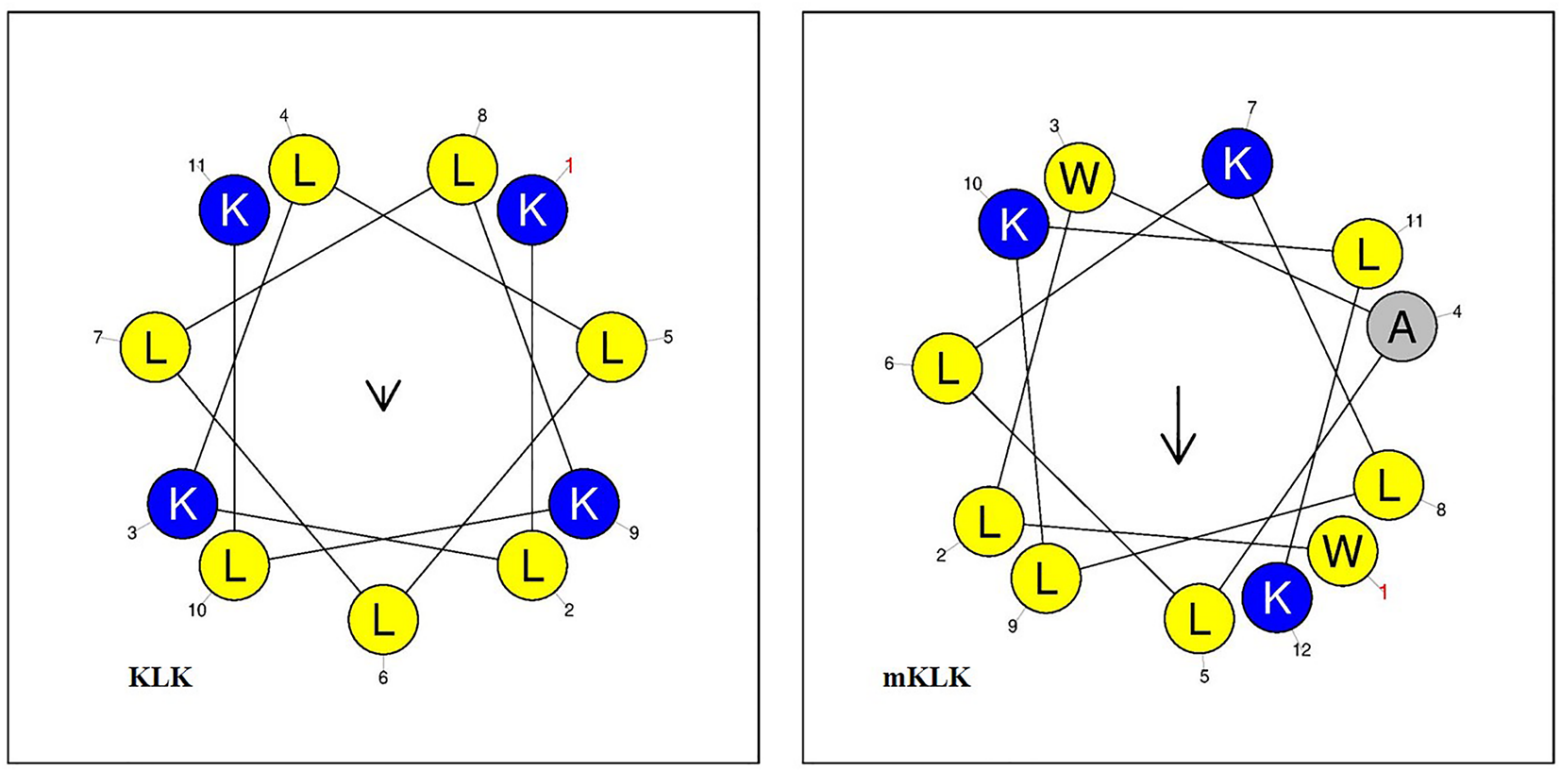
Helical wheel projections of KLK and mKLK. Helical wheels were drawn using the web server Heliquest (https://heliquest.ipmc.cnrs.fr/cgi-bin/ComputParams.py). A (alanine), L (leucine) and W (tryptophan) are hydrophobic and K (lysine) is hydrophilic residues. mKLK show superior amphipathicity (most of hydrophobic residues confined to one face of the helix) compared with the parent KLK peptide.

Peptide was designed based on findings of several studies^25,28^ and sequence-based analysis in the dPABBs database (http://ab-openlab.csir.res.in/abp/antibiofilm/)^29^. In comparison with the parent peptide (KLK), the KLK analogue (mKLK) possesses two Trp residues at positions 1 and 3, one alanine residue at position 4, and one lysine residue at position 7. Helical wheel projection of KLK and mKLK peptides showed that mKLK displayed superior amphipathic helixes (most hydrophobic residues confined to one face of the helix) compared with KLK (Fig. 1). The sequences and characteristics of KLK and mKLK are listed in Table 1.

**Table 1:** Physicochemical properties of KLK and mKLK.

Name	KLK	mKLK
Sequence	KLKLLLLLKLK-NH_2_	WLWALLKLLKLK-NH_2_
Net charge (pH7/pI)	+ 4	+ 3
Molecular weight (Da)	1323.02	1525.20
Hydrophobicity <H> ^a^	0.722	1.003
Polar residues; No. (%)	4 (36.4)	3 (25)
Nonpolar residues; No. (%)	7 (63.6)	9 (75)
Hydrophobic moment < µH > ^a^	0.095	0.324
Antibiofilm probability (SVM⃰)^b^	− 0.29	0.25

*SVM, support vector machine.

^a^Physicochemical parameter (hydrophobicity and hydrophobic moment) were calculated by HeliQuest, a web server to screen sequences with specific alphahelical properties [https://heliquest.ipmc.cnrs.fr/cgi-bin/ComputParams.py].

^b^AMP probability was predicted by a web-based prediction tool dPABBs (http://ab-openlab.csir.res.in/abp/antibiofilm/) using support vector machine (SVM) algorithm.

### MIC and MBC

As shown in Table 2, the values of MIC and MBC for KLK were equal to those of mKLK against all strains. KLK and mKLK exhibited relatively potent bactericidal activity against both MRSA and MSSA isolates. The MIC values against three MRSA and two MSSA isolates were 8 μg/ml and 4 μg/ml, respectively, indicating higher activity of both KLK and mKLK peptides against MSSA compared with MRSA strains. Moreover, the values of MIC and MBC for all isolates were equal. All isolates were susceptible to vancomycin (MIC = 0.25–0.5 μg/ml) (Table S2).

**Table 2:** The MIC, MBC, MBEC and SI values of the KLK and mKLK against *S. aureus* isolates.

Isolates	Biofilm formation abilities	Peptide KLK							Peptide mKLK						
		MIC		MBC		MBEC		SI	MIC		MBC		MBEC		SI
		μg/ml	μM	μg/ml	μM	μg/ml	μM		μg/ml	μM	μg/ml	μM	μg/ml	μM	
ATCC25923	Strong	4	3.03	4	3.03	512	387	7.275	4	2.62	4	2.62	64	42	19.45
MRSA-1	Strong	8	6.04	8	6.04	1024	774	3.63	8	5.24	8	5.24	64	42	9.725
MRSA-2	Strong	8	6.04	8	6.04	1024	774	3.63	8	5.24	8	5.24	128	84	9.725
MRSA-3	Moderate	8	6.04	8	6.04	1024	774	3.63	8	5.24	8	5.24	64	42	9.725
MSSA-1	Strong	4	3.03	4	3.03	512	387	7.275	4	2.62	4	2.62	32	21	19.45
MSSA-2	Strong	4	3.03	4	3.03	512	387	7.275	4	2.62	4	2.62	32	21	19.45

MIC, minimum inhibitory concentration; MBC, minimum bactericidal concentration; MBEC, minimum biofilm eradication concentration; SI, selectivity index calculated as the ratio between HC_50_ and MIC.

### Time-kill kinetics assays

**Figure 2:**
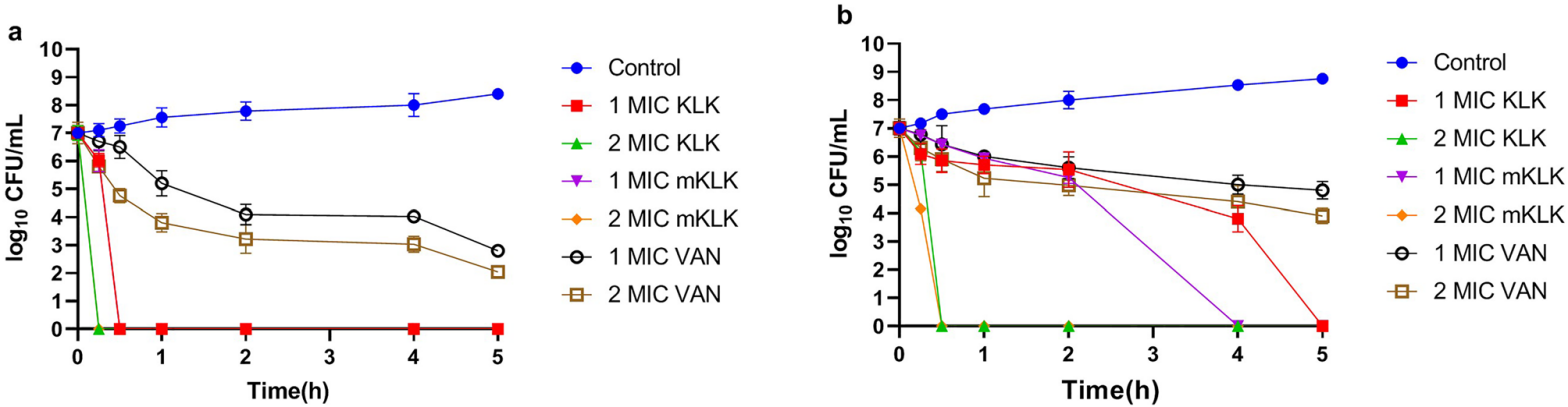
Time-Kill Kinetics of KLK, mKLK and vancomycin at 1× MIC and 2× MIC against ATCC 25923 (**a**) and MRSA isolate (**b**).

The time-kill kinetics of KLK and mKLK peptides at 1 × MIC and 2 × MIC against reference strain ATCC 25923 were compared with those of vancomycin (Fig. 2a). At 1 × MIC (4 μg/ml), mKLK caused approximately a 1-log reduction in viable cell count of ATCC 25923 during 15 min no regrowth was observed after 30 min. The time-kill kinetics of KLK at 1 × MIC (4 μg/ml) against ATCC 25923 was similar to mKLK and no growth was observed after 30 min. At 1 × MIC (0.25 μg/ml) of vancomycin, a ~ 3-log reduction in viable cell count was observed during 4 h; this slow decrease continued after 5 h, and no regrowth was observed after 24 h. Results of the time-kill kinetics assay at 2 × MIC (8 μg/ml) against ATCC 25923 showed that the bactericidal activity of mKLK was fast and there was no regrowth after 15 min. The bactericidal activity of KLK at 2 × MIC (8 μg/ml) against ATCC 25923 was similar to mKLK peptide and no growth was observed after 15 min. Vancomycin at this concentration (2 × MIC) caused a ~ 4-log reduction within 4 h; this slow reduction continued after 5 h, and no regrowth was observed after 24 h (Fig. 2a).

Time-kill kinetics of the peptides against MRSA strain was as follows: the mKLK peptide at 1 × MIC (8 μg/ml) led to a 2-log reduction in viable cell count of MRSA within 2 h and no regrowth was observed after 4 h, whereas the KLK peptide at 1 × MIC (8 μg/ml) caused a ~ 1-log reduction in viable cell count of MRSA within 2 h and no regrowth was observed after 5 h. At 1 × MIC (0.5 μg/ml) of vancomycin a slow decrease (~ 2-og) was detected for MRSA during 5 h and no growth was observed after 24 h. The time-kill kinetics of mKLK and KLK peptides at 2 × MIC (16 μg/ml) against MRSA strain were similar and no regrowth was observed within 30 min for both peptides. Moreover, at 2 × MIC (1 μg/ml) of vancomycin, approximately 2- and 3-log reductions were observed within 4 and 5 h, respectively; there was no growth after 24 h (Fig. 2b).

### Hemolytic activity of KLK and mKLK

**Figure 3:**
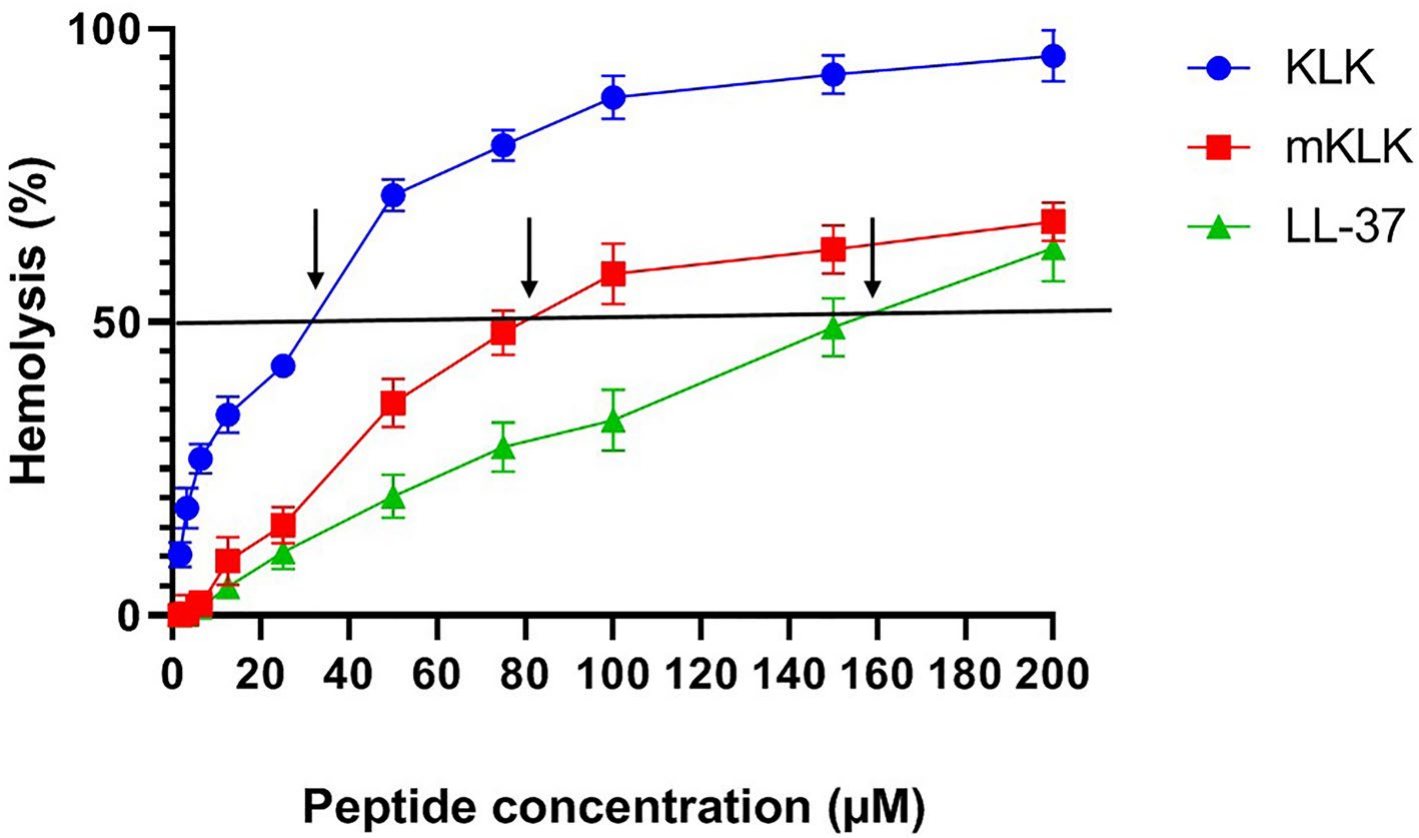
Hemolytic potency of the tested peptides (KLK, mKLK and LL-37) as a function of peptide concentration. The solid black line indicates 50% hemolysis, and the arrows indicate the HC_50_ values.

Hemolysis percent in samples is interpreted as follows: < 2%: non-hemolytic, 2–5%: slightly hemolytic, and > 5% hemolytic samples^30^. The mKLK peptide exhibited no hemolytic activity at concentrations from 1.56 µM (2.38 µg/ml) to 6.25 µM (9.531 µg/ml) and had hemolytic activity at concentrations from 12.5 µM (19 µg/ml) to 200 µM (305 µg/ml), whereas KLK was found to exhibit hemolytic activity at concentration from 1.56 µM (2.06 µg/ml) to 200 µM (264.6 µg/ml). Accordingly, mKLK had no hemolytic activity at concentrations corresponding to its MIC/MBC (4–8 µg/ml), while KLK exhibited hemolytic activity at MIC/MBC (4–8 µg/ml) (Fig. 3, Table S3). Moreover, The HC_50_ values were 77.8 µM (118.6 µg/ml), 29.1 µM (38.49 µg/ml), and 150 µM (673.5 µg/ml) for mKLK, KLK and LL-37, respectively. The SI values were observed in the range of 9.725–19.45 and 3.63–7.275 for mKLK and KLK, respectively (Table 2).

### Cytotoxicity of KLK and mKLK

**Figure 4:**
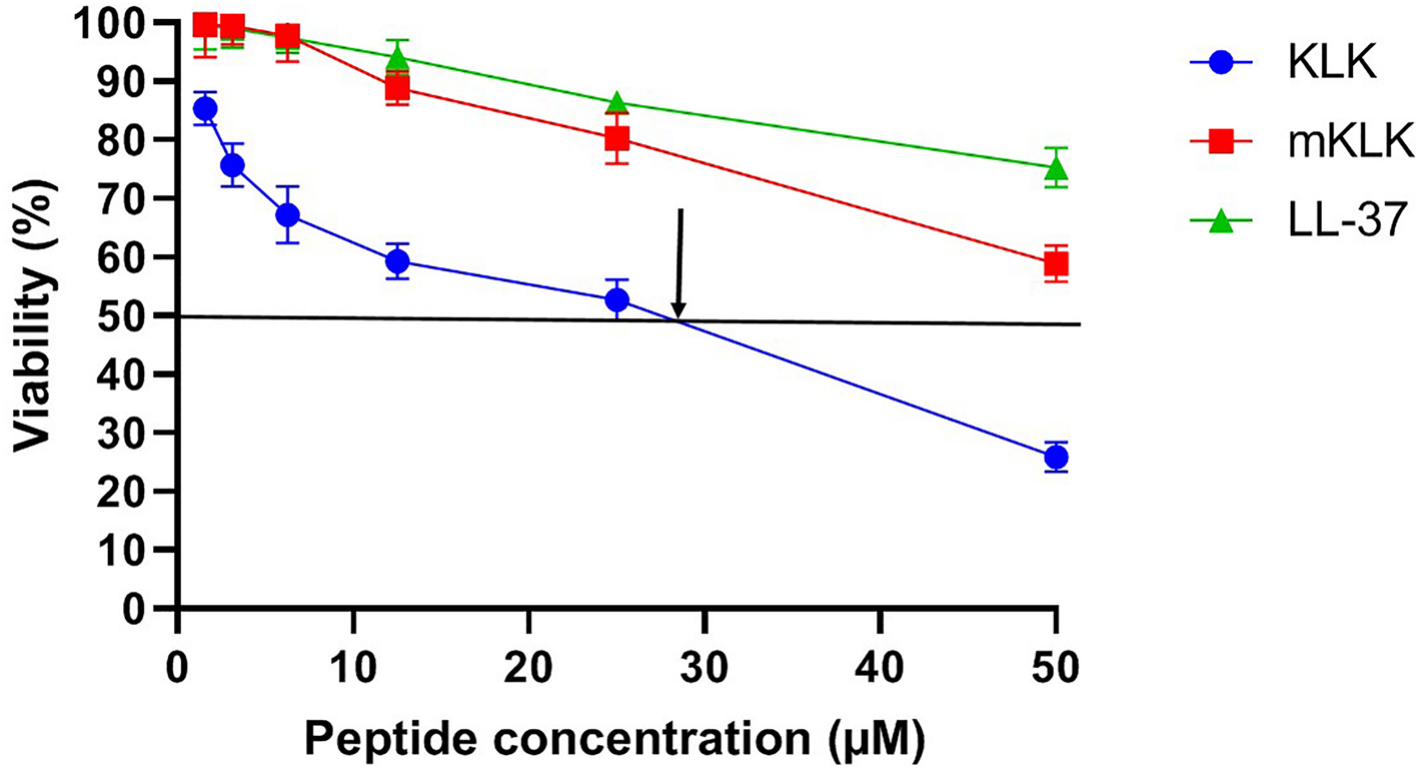
Cell viability of HEK-293 cell line at the different concentrations (1.56, 3.12, 6.25, 12.5, and 25 µM) of peptides (mKLK, KLK and LL-37) using XTT assay. The solid black line indicates 50% cytotoxicity, and the arrow indicates the IC_50_ values.

In the present study, the cytotoxic effects of different concentrations of KLK and mKLK were investigated using the XTT assay on the HEK-293 cell line. The results of cytotoxicity indicate that hemolysis were corresponded with cytotoxicity, so that at concentrations from 1.56 to 6.25 µM, mKLK had no significant toxic effect on the viability or morphology of the HEK-293 cell line compared with the control group (P > 0.05) but exhibited cytotoxic activity at concentrations from 12.5 (19 µg/ml) to 25 µM (38.25 µg/ml). KLK was found to exhibit cytotoxic activity at concentrations from 1.56 (2.06) to 25 µM (33 µg/ml). The IC_50_ value was calculated for KLK peptide 28.8 mM. The KLK SI value (IC_50_/MIC) was determined in the range of 3.6–7.2 (Fig. 4).

### Serum stability

Serum stability of the D-form mKLK peptide was considered as a percentage of the peptide remaining after incubation in 50% human serum solution at 37 °C for 2 h. Results of analysis by reverse-phase HPLC showed that 79% of the initial concentration of the mKLK peptide was retained after incubation in 50% human serum, suggesting the stability of the peptide structure to proteolytic degradation. Furthermore, following treatment of the D-form mKLK peptide with different serum concentrations (25%, 50%, and 90%) for 2 h, MIC of the peptide against *S. aureus* ATCC 25923 was determined to be equal to MIC of the serum untreated peptide (4 µg/ml), indicating retinue of its antimicrobial activity under human serum proteolytic digestion conditions.

### Quantification of biofilm using colorimetric assay and the viable plate count method

Based on the crystal violet assessment of the biofilms, strains were categorized into strong (OD570 ≥ 1.6), moderate (0.8 < OD570 < 1.6), or weak (0.4 < OD570 < 0.8) biofilm producers. As shown in Table 2, two MRSA, two MSSA, and ATCC25923 strains were strong and one MRSA strain (MRSA-3) was a moderate biofilm producer.

The plate count method was used to enumerate cultivable biofilm cells. The results of the plate count corresponded to the crystal violet assay. The count of *S. aureus* cells ranged from 6.9 ± 0.12 to 7.3 ± 0.17 Log CFU/well for strong biofilm formers and 6.4 ± 0.24 for moderate biofilm former. There were no significant differences in quantities of biofilm between MSSA and MRSA strains (P > 0.05).

### Antibiofilm activity

**Figure 5:**
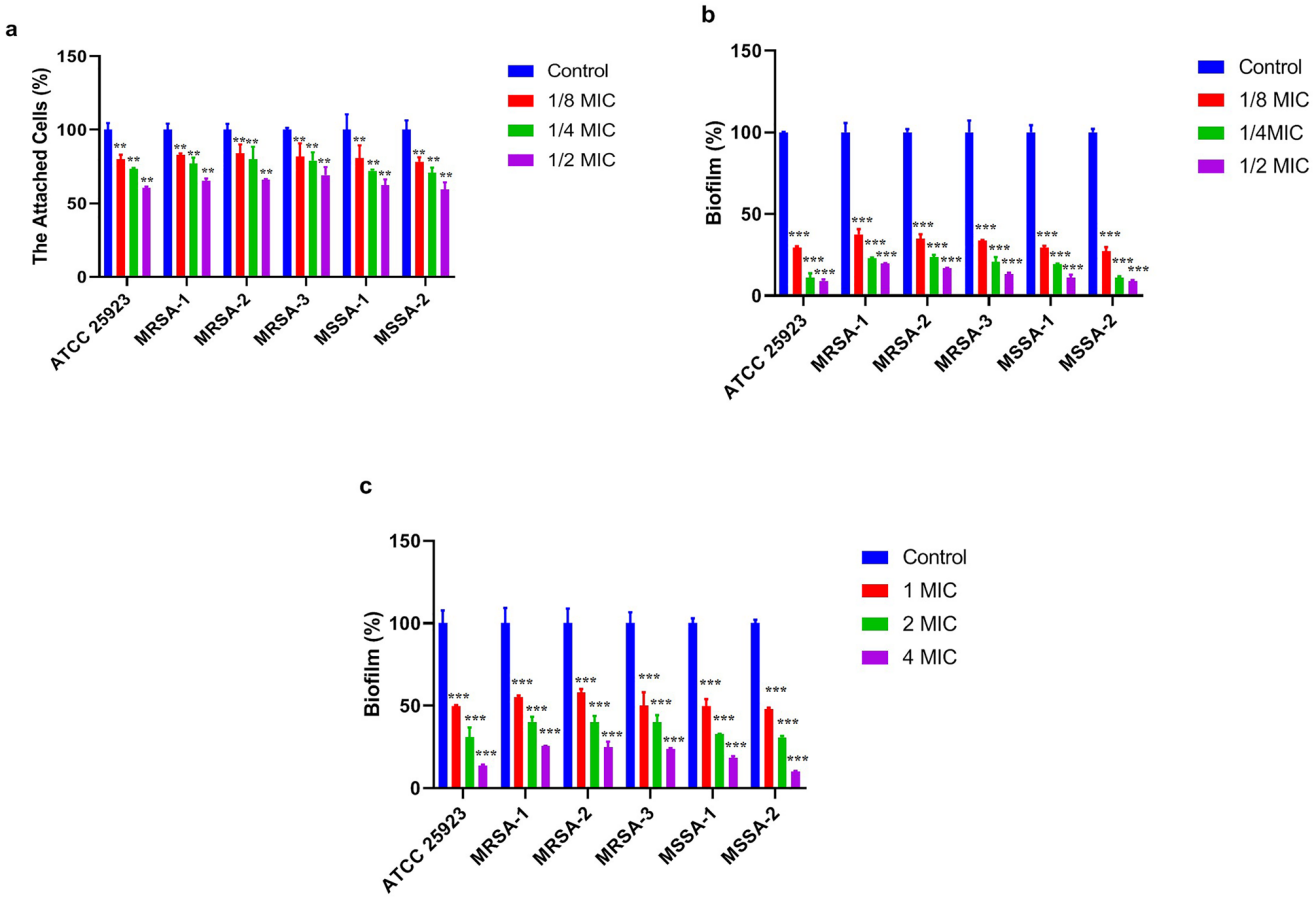
Each bar represents the Mean ± SD formed biomass undergoing treatment by mKLK peptide at 1/8×, 1/4× and 1/2× MIC. Anti-biofilm properties of. Adhesion inhibitory activity (**a**); biofilm inhibitory activity (**b**); biofilm dispersal activity at 1×, 2× and 4× MIC (**c**); *P < 0.05; **P < 0.01, ***P < 0.001.

**Figure 6:**
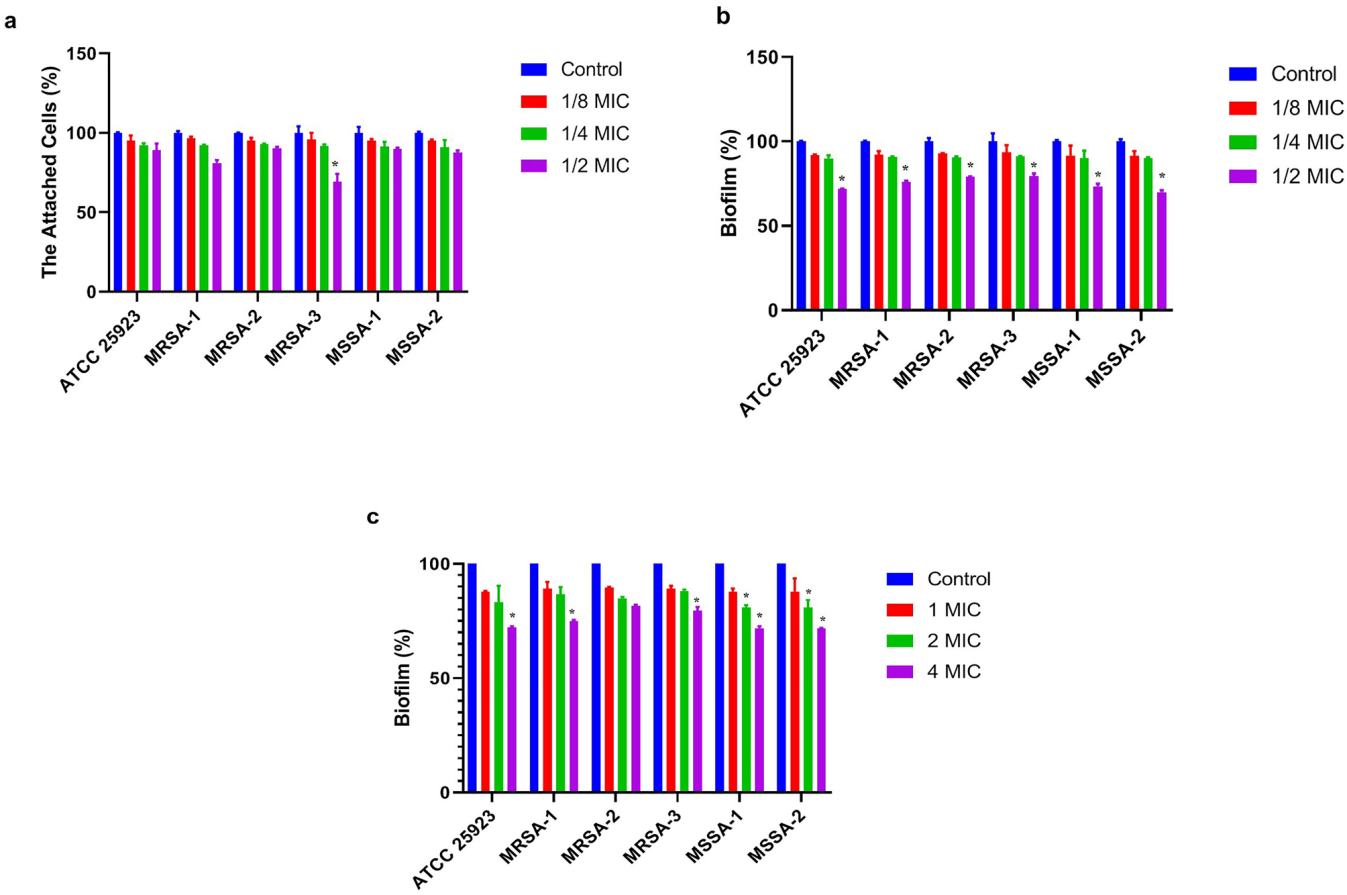
Each bar represents the Mean ± SD formed biomass undergoing treatment by KLK peptide at 1/8×, 1/4× and 1/2× MIC. Anti-biofilm properties of. Adhesion inhibitory activity (**a**); biofilm inhibitory activity (**b**); biofilm dispersal activity at 1×, 2× and 4× MIC (**c**); *P < 0.05; **P < 0.01, *** P < 0.001.

Results revealed that the mKLK peptide at 1/8×, 1/4×, and 1/2× MIC significantly caused inhibition of initial adhesion of all strains by 16–22%, 20–29%, and 31–41%, respectively (Fig. 5a, Table S4, P ≤ 0.01). The adhesive inhibitory effect of KLK on all strains was not significant. The adhesion inhibition rates of *S. aureus* strains by KLK at 1/8×, 1/4×, and 1/2× MIC were 4–5%, 7–9%, and 11–31%, respectively (Fig. 6a, Table S4).

Following incubation for 24h, the mKLK peptide at 1/8×, 1/4×, and 1/2× MIC significantly inhibited the biofilm formation by 63–73%, 76–89%, and 80–91%, respectively (P < 0.001) (Fig. 5b and Table S5). Compared with KLK, the mKLK peptide exhibited a higher inhibition ability of the biofilm formation in all strains (Fig. 6b and Table S5).

The highest biofilm inhibitory activity of the KLK peptide was observed at 1/2× MIC with an inhibition rate ranging from 48 to 66% (P ≤ 0.05). Moreover, following incubation for 24 h, the KLK peptide at 1/8× and 1/4× MIC inhibited the biofilm formation in all strains by 6–9% and 9–11%, respectively (Fig. 6b and Table S5).

The mKLK peptide significantly dispersed the 24 h pre-formed biofilm at 1×, 2×, and 4× MIC by 42–52%, 60–70%, and 75–90%, respectively (P < 0.001) (Fig. 5c, Table S6), whereas KLK exhibited a lower dispersal activity at these concentrations. Dispersion rates of *S. aureus* biofilms by KLK at 1×, 2×, and 4× MIC were 10–12%, 17–20%, and 18–29%, respectively (P ≤ 0.05; Fig. 6c, Table S6).

The mKLK peptide eradicated the pre-formed biofilm cells at concentrations from 32 to 128 μg/ml (MBEC = 32, 64, and 128 μg/ml), whereas KLK exhibited eradicative activity at concentrations from 512 to 1024 μg/ml (MBEC = 512–1024 μg/ml) (Table 2).

### In vivo inhibitory effect of mKLK on biofilm formation

**Figure 7:**
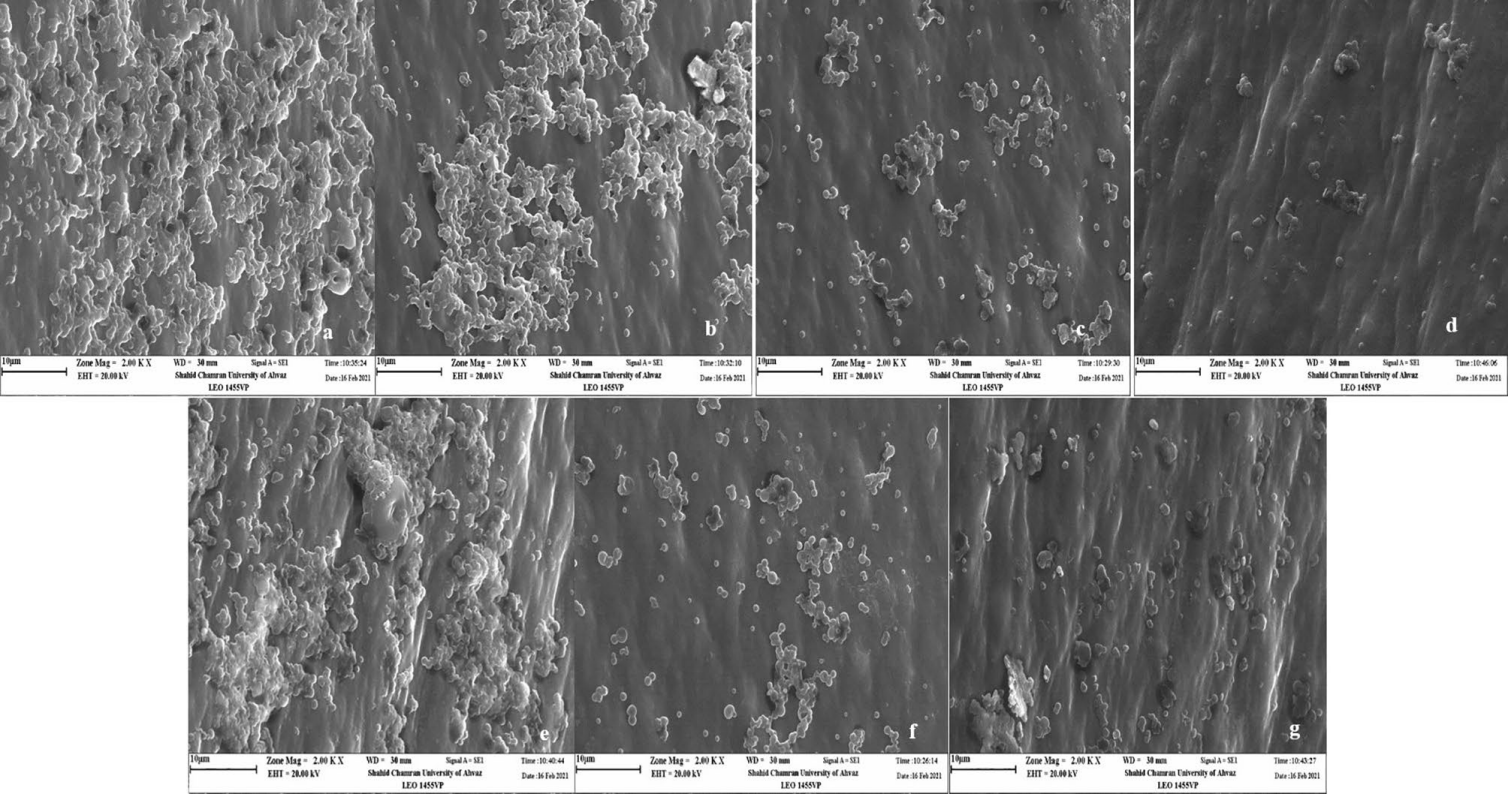
Images of *S. aureus* biofilm on catheters after treated with KLK peptide (Scale bar 10 μm). (**a**) Biofilm of control group without treatment, (**b**) Biofilm of ATCC 25923 after treatment with 1/×4MIC, (**c**) Biofilm of ATCC 25923 after treatment with 1/2 × MIC, (**d**) Biofilm of ATCC 25923 after treatment with 1× MIC, (**e**) biofilm of MRSA after treatment with 1/4× MIC, (**f**) biofilm of MRSA after treatment with 1/2× MIC, (**g**) biofilm of MRSA after treatment with 1× MIC.

**Figure 8:**
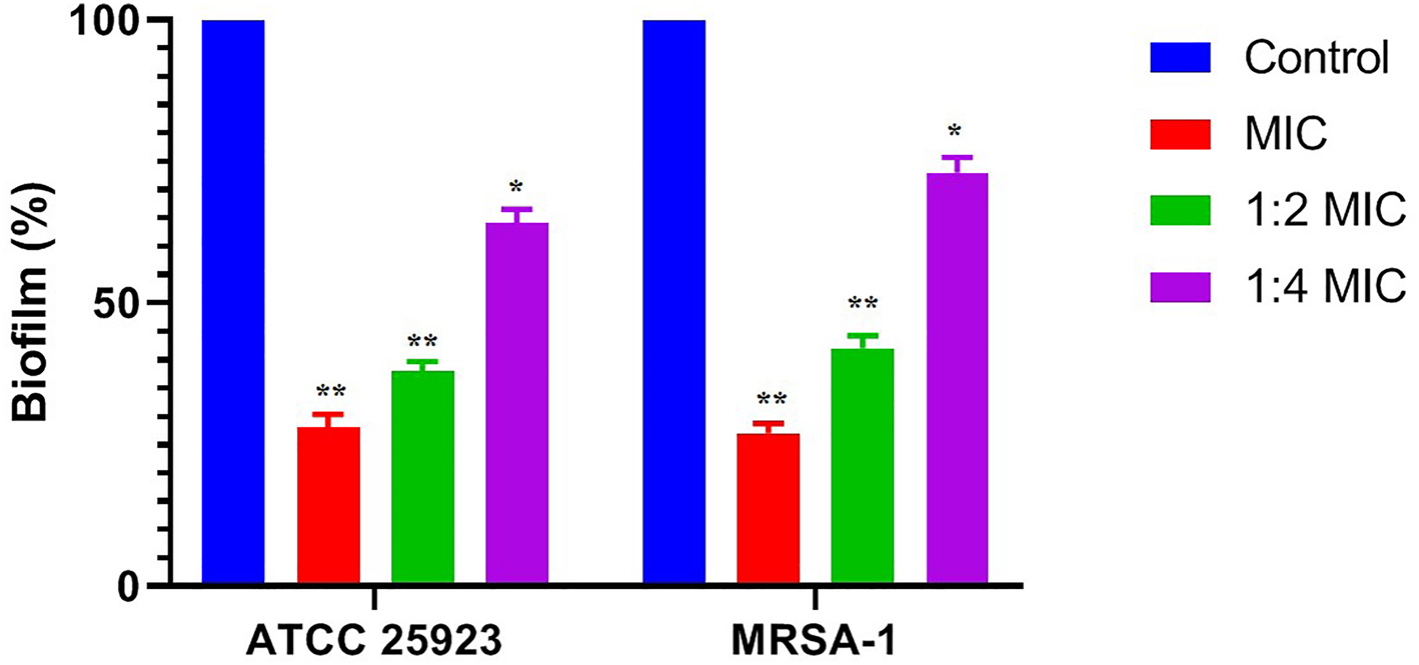
In vivo biofilm mass formed on the catheters after treatment using different concentration (1 MIC, 1/2 MIC and 1/4 MIC) of mKLK peptide by crystal-violet assay. *P < 0.05; **P < 0.01, ***P < 0.001.

The biofilm inhibitory effect of mKLK against two different strains of *S. aureus* (*S. aureus* ATCC25923 and MRSA-1 strains) was investigated using a catheter-associated infection mouse model. As seen in the SEM images, a denser biofilm was observed in the untreated catheters compared with ones treated with 1/2×, 1/4×, and ×1 MIC of mKLK for both *S. aureus* strains (Fig. 7a–g). Visual comparison of the catheters SEM images showed that there is fewer bacterial biofilm cells on catheters treated with 1× and 1/2× MIC of mKLK compared with one treated with 1/4× MIC of the peptide, which corresponded to the result of crystal violet and plate count methods. Result of the crystal-violet assay showed that the biofilm formation in ATCC 25923 was significantly reduced by 36%, 62%, and 72% at 1/4×MIC, 1/2× MIC, and 1 × MIC of mKLK, respectively (P < 0.05) (Fig. 8). In addition, MRSA biofilm was significantly reduced by 27%, 58%, and 73% at 1/4×, 1/2× MIC, and 1× MIC of mKLK, respectively (P < 0.05) (Fig. 8). Results of the plate count method showed a CFU count of 7.9 log_10_ ± 0.89 CFU/catheter for the untreated control, and 7.68 log_10_ ± 0.43, 7.44 log_10_ ± 0.64 and 7.30 log_10_ ± 0.13 CFU/catheter for catheters treated with 1/4×, 1/2× and 1× MIC of mKLK, respectively, in the reference strain ATCC 25923. In the MRSA strain, catheters treated with mKLK at 1/4×, 1/2×, and 1× MIC led to a CFU count of 7.72 log_10_ ± 0.11, 7.49 log_10_ ± 0.38, and 7.28 log_10_ ± 0.38 CFU/catheter, respectively. According to the results mentioned above, the results of the plate count method corresponded to the ones of the crystal violet assay.

## Discussion

The additional worrisome factor related to *S. aureus* pathogen is its potential to cause biofilm-associated infections. *S. aureus* colonizes the surface of medical equipment and medical devices to form biofilms, leading to antibiotics-resistant long-term and recurrent infections in patients^31^. Accordingly, there is an urgent need for novel therapeutic drugs able to kill not only planktonic cells but also biofilm ones. Drug resistance and toxicity of conventional antibiotics led to the discovery of many AMPs as natural anti-biofilm agents^32^.

In the present study, the antibacterial and antibiofilm activities of a novel analogue peptide (mKLK) based on an amidated synthetic sapecin B-derived peptide (KLK) were evaluated against clinical strains of MRSA and MSSA. Although the bacteriocidal activity of both D and L-form of KLK, and its variants including KLKLLLLLKL (KLK_1_) and KLKLLLLLK (KLK_2_) was investigated in previous studies^17,27,33^, there are no studies to evaluate antibiofilm potency of these peptides. To the best of our knowledge, this is the first research to investigate the antibiofilm potency of the KLK peptide in comparison to its novel analogue (mKLK) with antibiofilm activity. Furthermore, the mKLK peptide is the only reported KLK analogue that possesses antibiofilm potency.

The mKLK peptide has two hydrophobic aromatic amino acids tryptophan at the N-terminus, while the parent KLK, KLK_1_, and KLK_2_ have no tryptophan. Several researchers have declared that variants of individual AMPs with enhanced cationic, especially the one coming from an arginine or stretched N-terminus with hydrophobic aromatic amino acids (tryptophan or phenylalanine) are related to increased antibacterial activity^34–36^. It is possible that an increase in the positive net charge of the peptides results in stronger attachment to the negatively charged composition on the bacterial surface, allowing more peptide molecules to assemble on the bacterial membrane and consequently be more effective toward bacteria^37,38^. Although the positive charge of mKLK is less than KLK, the reduction in cationicity may have been offset by a slightly higher hydrophobicity and superior amphipathic helixes for mKLK. Helical wheel projection shows that mKLK displayed superior amphipathic helixes in which the most of hydrophobic residues confined to one face of the helix compared with KLK (Fig. 1). Two lysine residues at the N-terminus of the parent peptide KLK (position 1 and 3) were replaced by two tryptophan residues (WLWALLKLLKLK). Several studies revealed that the arrangement of tryptophan residues at the N-terminus of the peptides had a significant influence on enhancing the antibacterial efficacy of AMP, whereas the arrangement of the C-terminal tryptophan residues not only has little effect on the antibacterial activity but also increases the toxicity of the peptide to blood cells and mammalian cells^25–28^.

We demonstrated that mKLK at different concentrations (5–100 μg/ml) has no significant hemolytic activity toward human red blood cells. MSSA clinical isolates and reference strain ATCC 25923 in planktonic mode exhibited susceptibility to mKLK with a MIC of 4 μg/ml. In addition, the mKLK MIC against MRSA clinical isolates which additionally were resistant to vancomycin was found to be 8 mg/ml. Manabe et al.^17^, evaluated the antimicrobial activities of both D-form and L-form of KLK against bacteria, including *S. aureus*, *Candida albicans*, and *Escherichia coli*. The MIC values of D-form were 4 μg/ml and L-form was > 128 for *S. aureus*. In contrast, in another study, the antibacterial activity of KLK and KLK1 was evaluated toward clinical Salmonella isolates. The results of this study showed that the tested peptides have no antibacterial activity with a MIC > 256 µM^27^. In another study by Sudadech et al.^33^, in vitro activities of S61, S62, S63, and KLK1 were investigated against drug resistant *Mycobacterium abscessus* isolates. They stated that all four AMPs exhibited ~ 99% killing against 62.5% of the tested isolates within 24 h with a MIC < 50 μg/ml. In the mentioned studies, the activity of different variants of KLK was analyzed.

The time killing kinetics of mKLK and vancomycin, as a selective antibiotic, against MRSA and reference strains were also compared with the parent KLK. Results revealed lack of bacterial regrowth after treatment with peptide at 1× MIC in the early hours (30 min for reference strain and 4 h for MRSA isolates). At a higher concentration (2× MIC), the peptide rapidly showed its bactericidal effect, which is a favorable effect in the fight against severe infections. In contrast, antibacterial activity of vancomycin was slower and accrued over a 24 h period. This difference in activity rate between mKLK and vancomycin can be related to their different mechanism of action. However, a definite statement requires more detailed pharmacodynamics studies. In the current study, equality of MIC and MBC and the lack of bacterial growth after treatment with the peptide in the early hours are evidences of bactericidal activity of mKLK. It seems that mKLK can limit *S. aureus* infection in the early hours of bacterial colonization.

In this study antibiofilm activity of mKLK is investigated. This potential was examined from four aspects as follows: inhibitory effect on bacterial attachment, inhibitory effect on biofilm formation, eradication of bacteria in biofilm mode (MBEC), and dispersal of preformed biofilm matrix. Findings of antibiofilm activity assessment indicated that the mKLK peptide had a significant inhibitory effect on bacterial attachment to surfaces and biofilm formation, as well as a significant dispersal activity at sub-MIC concentrations (1/8×, 1/4×, and 1/2 × MIC) in a dose-dependent manner. As results shown, sub-MIC concentrations of the tested peptide caused a reduction in bacterial attachment of all investigated isolates. These concentrations also considerably inhibited the biofilm formation of all isolates after 24 h incubation.

To ensure that the antibiofilm property of mKLK is independent of its ability to inhibit bacterial growth, the effect of 1/2× MIC on bacterial growth was examined. The result demonstrated that the peptide at this concentration did not affect the bacterial growth and thus, antibiofilm activity does not result from inhibition of bacterial growth.

Results of MBEC determination showed that higher concentrations (3× MIC to 4× MIC) than sub-MICs are required for the eradication of preformed biofilm. This finding showed the ability of the peptide to penetrate in biofilm matrix and to affect bacteria in biofilm mode.

One of the important aspects of catheter-related infections is biofilm formation. Catheter replacement surgery is usually the only option available for treatment. The most efficient methods to prevent catheter-associated infections are to avoid unnecessary catheterizations and to remove catheters as soon as possible^39^. Accordingly, the antibiofilm activity of mKLK was further confirmed on catheter-associated infection using a mouse model. Our finding revealed that mKLK significantly inhibited biofilm development and as a result, reduced biofilm on the mouse catheter at 1/4×, 1/2×, and 1× MIC concentrations of mKLK. In vivo investigation of mKLK has not been performed up to now. AMPs can inhibit biofilm formation using the following ways; (1) interference with quorum sensing signaling, (2) inhibition of the alarm system to suppress intense bacterial reactions, and (3) reduction of the expression of binding protein-dependent transport system genes associated with biofilm formation^40^. These data emphasize that the mKLK peptide could exert antibiofilm activity against reference (ATCC 25923) and clinical (MSSA and MRSA) strains of *S. aureus* by inhibiting initial bacterial attachment and destroying preformed biofilms.

In summary, the obtained MIC and MBEC values indicated a strong effect of the mKLK peptide on *S. aureus* in planktonic and biofilm modes without cytotoxicity. Moreover, the inhibitory effect of sub-MIC concentrations of the peptide on bacterial attachment and biofilm formation demonstrated that mKLK can be developed as a novel therapeutic drug for preventing colonization, onset of biofilm formation in catheter-associated *S. aureus* infections, and for treating planktonic and biofilm-associated drug-resistant *S. aureus* infections.

## Conclusion

In conclusion, in the present study, for the first time, a novel analogue peptide (mKLK) designed based upon an D-form amidated synthetic sapecin B-derived peptide (KLK) was shown to display a potent antimicrobial and antibiofilm activity against MRSA and MSSA. Furthermore, the inhibitory activity of the peptide at sub-MIC concentrations on bacterial attachment and biofilm formation indicated that mKLK could be developed as a promising therapeutic drug for preventing biofilm-associated MRSA and MSSA infections. Recent approaches for peptide immobilization offer reliable possibilities for the prevention of biofilm infections on medical devices and represent an attractive concept for immobilizing antibiofilm peptides such as mKLK.

## Materials and methods

### Ethics approval and consent to participate

The present research was approved by the ethics committee of Ahvaz Jundishapur University of Medical Sciences (Ethical code: IR.AJUMS.ABHC.REC.1400.014). All experiments in this study were performed in accordance with protocols and regulations approved by ARRIVE guidelines (https://arriveguidelines.org).

### Bacterial strains

Three MRSA, two MSSA clinical isolates, and one *S. aureus* reference strain (ATCC 25923) were investigated in this study. In order to confirm MRSA and MSSA isolates, antimicrobial susceptibility testing (AST) was performed for 12 antibiotics and to cefoxitin (Mast, UK) by Kirby-Bauer disk diffusion method on Mueller–Hinton agar (MHA) according to Clinical and Laboratory Standards Institute (CLSI) guidelines (Table S1)^41^. The presence of the mecA/mecC gene was studied by PCR in all cefoxitin resistant isolates^42,43^.

### Peptide synthesis

The D-form amidated KLK peptide and D-form amidated mKLK peptide (purity ≥ 95%) were purchased from Proteomics International laboratories (Proteomics International Laboratories Ltd., Australia).

### Time-kill kinetics assay

In the present study, a time-kill kinetics assay was used to investigate the killing rate of mKLK and KLK peptides against one MRSA and reference strain (ATCC25923) in comparison with vancomycin. For each strain, a suspension with a final density of 10^7^ CFU/ml was prepared from overnight cultures and added to each well of the 96-well microtiter plate in the presence of peptides (1×, and 2× MIC). The peptide-free control was included for each strain. Following incubation at 37 °C for 0, 0.25, 0.5, 1, 2, 4, 5, and 24 h, each sample was serially diluted, plated onto Mueller–Hinton (MH) agar (Merck, Germany) plates and incubated at 37 °C. The viable plate count method was performed after incubation for 24 h and the counts were expressed as Log CFU/ml^44^.

### The hemolysis assay

The hemolysis assay was used to evaluate the toxicity of mKLK and KLK peptides in comparison with the LL-37 peptide. The assay was performed using fresh human blood samples containing citrate phosphate dextrose anticoagulant collected from a blood bank (Iranian Blood Transfusion Organization). Human red blood cells (RBC) were centrifuged at 1700×g for 5 min; then the supernatant was removed and the erythrocyte pellet was diluted 1:100 in PBS to obtain a 1% erythrocyte suspension. The tested peptides (KLK, mKLK, and LL-37) were prepared with the Tris-saline buffer at different concentrations (1.56, 3.12, 6.25, 12.5, 25, 50. 75, 100, 150, and 200 μM). Then, 50 μl of the 1% erythrocyte suspension and 50 μl of the peptides were mixed and added to wells of a 96-well polypropylene conical bottom microplate and incubated at 37 °C for 1 h. Following centrifugation of the samples at 1700×g for 5 min, 50 μl of the supernatant was transferred to wells of a 96-well flat-bottom plate for the absorbance measurement at 405 nm in a microplate reader. Identical volumes of Triton X-100 (1%) and PBS were used as positive and negative controls, respectively^45^. The selectivity indexes (SI) were also determined as a toxicity indicator. SI is defined as the ratio between the concentration leading to 50% lysis of human erythrocytes (HC_50_) and the minimum concentration inhibiting bacterial growth (MIC) (SI = HC_50_/MIC), which is sometimes also considered a ‘therapeutic index’^46^.

### Cytotoxicity assay

To quantitatively investigate the cytotoxicity of mKLK and KLK peptides, the 2,3-bis-(2-methoxy-4-nitro-5-sulfophenyl)-2H-tetrazolium-5-carboxanilide (XTT) (cell proliferation kit II, Roche, Germany) assay was performed on human embryonic kidney (HEK)-293 cell line, as described previously^47^, The HEK-293 cells were grown in DMEM supplemented with 4 µM glutamine, 10% fetal bovine serum (FBS), and 100 units/ml penicillin/streptomycin at 37 °C in a 5% CO_2_ and 95% air atmosphere. Following incubation for 24 h, the culture medium was discarded and fresh medium containing different concentrations of KLK and mKLK peptides (1.56, 3.12, 6.25, 12.5, and 25 µM) was added and incubated at 37 °C for 20 min. Then, the culture medium was discarded and 100 µl of XTT reagent mixture was added and incubated for 4 h. Finally, the absorbance was measured at 570 nm using an ELISA plate reader (Anthos 2020, England). The percentage of cell inhibition was calculated using the following formula: % Cell inhibition = Test Abs/Control Abs × 100. For cytotoxicity assay, the selectivity index (SI = IC_50_/MIC) of the peptides was also determined.

### Stability of peptides in human serum

To assess the serum stability of mKLK peptide to proteolytic degradation, the D-form mKLK peptide (0.5 mg) was dissolved in 4 ml of 50% (v/v) of serum from male healthy donors in PBS and incubated at 37 °C for 0, 60, and 120 min. After each incubation time, the samples were analyzed by reverse-phase HPLC on a Jupiter proteo C18 column (250 × 4.6 mm) and monitored with a fluorescence detector (wavelength: 560 nm). Analysis of the samples was performed over a linear gradient of 10–60% acetonitrile containing 0.08% trifluoroacetic acid for 50 min at a flow rate of 1 ml/min^48^.

To test the retinue of antimicrobial activity of mKLK in human serum, MIC of the serum-treated peptide was determined against *S. aureus* ATCC 25923 using microdilution broth assay. The various concentrations of mKLK (0.5, 1, 2, 4, 8, 16, 32, 64, and 128 μg/ml) were incubated in 96-well microtiter plates at different concentrations of serum (25%, 50%, and 90%) at 37 °C. The following incubation for 120 min, bacterial suspensions (5 × 10^5^ CFU/ml) were added to the mixtures and incubated at 37 °C for 20 h. The MIC was determined as the lowest concentration of peptide at which no visible growth is seen^49^.

### MIC and MBC determination

Minimum inhibitory concentrations (MIC) of KLK, mKLK, and vancomycin were determined using microdilution broth assay according to clinical and laboratory standards institute (CLSI) guidelines with some modifications^41^. Briefly, overnight cultures were diluted in Muller-Hinton broth (MHB; Merck, Germany) to achieve a final density of 5 × 10^5^ CFU/ml. A volume of 100 μl of bacterial suspension was added to wells of sterile 96-well plates containing 100 μl of two-fold peptide dilutions, ranging from 0.25 to 256 µg/ml. Vancomycin was used as a positive control in this assay. After incubation at 37 °C for 18 h, the lowest concentration of peptide at which no visible growth is seen was defined as the MIC, while the lowest concentration of antimicrobial causing at least 99.9% killing of the initial inoculums was considered as MBC. MBC was determined by removing samples from wells with no visible growth, serially diluting, and plating on Mueller Hinton (MH) agar plates for CFU counting.

### Biofilm formation ability and quantification of biofilm

The biofilm formation capacity of isolates was evaluated using the microtiter plate method^50^. Briefly, One colony of the overnight culture of *S. aureus* on tryptic soy agar (TSA) was suspended in tryptone soy broth (TSB) medium and incubated at 37 °C, 125 rpm until an optical density at 600 nm (OD_600_ nm) of 0.1 was reached (approximately 8 Log_10_ CFU/ml). This inoculum was diluted 1:100 in fresh TSB medium to a concentration of 6 Log_10_ CFU/ml. An aliquot of 200 µl/well was transferred to a 96-well flat-bottom microtiter plate (Spl Co, South Korea) and incubated statically at 37 °C for 24 h in a humidified incubator. Wells containing only TSB were used as negative controls. Following incubation at 37° C for 24 h, the medium was discarded and the wells were washed thrice with phosphate-buffered saline (PBS). After fixation of the biofilm with methanol for 15 min, methanol was discarded and the wells were air-dried at room temperature and then stained with 0.1% crystal violet for 5 min. All wells were washed with water and air-dried. Then, acetic acid glacial 33% was added to each well and the plate was shaken for 30 min. Absorbance measurements at 570 nm were done by a spectrophotometer to quantify the biofilms. The following criteria were used to categorize the biofilm forming power of *S. aureus* strains to no biofilm producers, weak, moderate, or strong biofilm producers, as previously described^51,52^. Briefly, the cut-off OD (ODc) was defined as three standard deviations above the mean OD of the negative control. Strains were classified as follows: OD ≤ ODc = no biofilm producer, ODc < OD ≤ (2 × ODc) = weak biofilm producer, (2 × ODc) < OD ≤ (4 × ODc) = moderate biofilm producer and (4 × ODc) < OD = strong biofilm producer.

To enumerate cultivable biofilm cells, biofilms were developed in 24-well plates. Following static incubation at 37 °C for 24 h, the medium was discarded and the wells were washed thrice with PBS. After the addition of 100 μl of sterile PBS solution, the wells were thoroughly scraped with a sterile pipette tip, vortexed, and sonicated to release the biofilm cells from the well surface. The suspended biofilms were then serially diluted, plated onto TSA plates, and incubated at 37 °C. After incubation for 24 h, CFUs are counted on the plates for three independent experiments, and the number of cells per milliliter (CFU/ml) in the original cultures are calculated as the mean ± SD of colony counts.

### Anti-biofilm properties

To investigate the inhibitory effect of KLK and mKLK peptides on bacterial surface attachment and biofilm formation, the bacterial suspension in TSB with a concentration of 10^7^ CFU/ml was prepared from overnight culture. An aliquot of 100 μl of the bacterial suspension was added to wells of 96-well plates containing 100 μl of the peptides solution with concentrations of 1×, 1/2× and 1/4× MIC, resulting in the final peptides concentrations of 1/2×, 1/4× and 1/8× MIC. To investigate the inhibitory effect of the peptides on surface attachment the incubation was performed at 37 °C for 2 h and to evaluate their inhibitory activity on biofilm formation the incubation was performed at 37 °C for 24 h. The wells containing only TSB medium were used as negative controls. After incubation, the contents of the wells were removed and washed three times with PBS and the wells were stained with 200 μl of 0.1% crystal violet for 20 min. The plates were washed again and excess dye was removed. A volume of 200 μl of 95% ethanol was added to each well for 20 min and absorbance was measured at 570 nm by a microplate reader^53^.

### Minimum Biofilm Eradication Concentration (MBEC)

The eradicative activity of KLK and mKLK peptides against preformed *S. aureus* biofilms was evaluated by determining MBEC, as previously described^54^. The 24-h biofilms developed in TSB in a 96-well plate were washed three times with 200 μl PBS solution. Concentrations of the peptides ranging from 2 to 1024 µg/ml were prepared. A volume of 100 μl of each concentration was added to a corresponding well, and plates were incubated for 18–20 h at 37 °C. Then, wells were washed with sterile PBS solution, and 100 μl of PBS solution was added. The wells were thoroughly scraped with a sterile pipette tip, vortexed and sonicated to release the biofilm cells from the well surface. Subsequently, 10 μl of well contents were plated on MHA. Colonies were counted after 20 h at 37 °C. The MBEC was defined as the lowest concentration of peptide that inhibited bacterial regrowth.

### Dispersal activity

To investigate the dispersal activity of KLK and mKLK peptide, the 24-h *S. aureus* biofilms were prepared in TSB medium in the 96-well plates. Then, the final concentrations of 1×, 2×, and 4 × MIC were prepared for each peptide in the wells. After incubation at 37 °C for 12 h, plates were washed with PBS three times to discard unattached bacteria. Subsequently, plates were stained with 250 μl of 0.5% crystal violet for 20 min at room temperature. Then, the wells were washed and 250 μl 33% acetic acid was added to them. Finally, the absorbance was measured at 570 nm using a microplate reader^54^.

### In vivo antibiofilm activity of mKLK peptide

All animal experiments were performed in accordance with the protocols approved by the Animal Ethics Committee of Ahvaz Jundishapur University of Medical Sciences (Application No. IR.AJUMS.ABHC.REC.1400.014). Female *Balb/c* mice (Laboratory Animal Reproduction and Maintenance Center Ahvaz Jundishapur University Ahvaz, Iran) were housed one mouse per cage, under sanitary conditions at 22–25 °C and at 12 h light/dark cycles, with access to sanitized pellet food and water. Prior to each experiment, mice were acclimated to room conditions for 1 week. To increase the experimental accuracy, the cages were disinfected with a povidone-iodine solution of 10%, and the bedding materials were autoclaved, and replaced every day.

In this study, eight groups (five mice per group) of 12-week-old female *Balb*/c mice were obtained from the Pasture Institute of Iran (Tehran, Iran). Three groups were used to investigate the effect of three concentrations of mKLK against ATCC25923 strain, three groups for three concentrations of mKLK against MRSA, and two groups for control). Mice were anesthetized by injection of ketamine 10% and xylazine 2% (in a ratio of 3 to 1). To create a subcutaneous pocket to place three 1-cm-long polyurethane catheters, a small incision was made. Three catheters were subcutaneously implanted on the lower back of mice. Following implantation of the catheters, injection of bacterial suspension and then mKLK peptide was performed into the subcutaneous pocket. The total volume of injection was 250 µl in which the final bacterial concentration was 10^6^ CFU/mL and the final peptide concentration was 1×, 1/2×, or 1/4× MIC. In addition, PBS was injected alone into animals as a control group without contamination to evaluate the sterility of the surgical procedure. The incision was closed with Vetbond. Tissue adhesive (Kimiatajhiz teb, Iran) and cleansed with 10% PI solution. After 5 days, all mice were euthanized with CO_2_, and the catheters were removed^55^. Subsequently, catheter-associated biofilms were evaluated using the CFU counting method, crystal violet assay, and SEM.

### Evaluation of biofilm on catheters

The biofilm mass attached to the catheter surface was measured using a colorimetric assay (crystal violet). After removing the catheters from the mouse skin and double washing with PBS, each catheter was placed in wells of a 24-well microtiter plate, and absolute methanol was added to fix the biofilm to the catheter surface. Then, the methanol was discarded and the catheters were dried at room temperature. After staining with crystal violet 0.1% for 5 min at room temperature, the dye was discarded and the catheters were washed with PBS. Then, 33% acetic acid was added to each well to release the absorbed dye. Finally, absorbance was measured at 570 nm and results were compared with the control. To enumerate biofilm cells, catheters were removed, transferred to sterile tubes, vortexed, and sonicated. Bacterial numbers were determined by serial diluting and plating on TSA^56^.

### Scanning electron microscopy

To observe the biofilm mass on the catheters, the catheters removed from the mice's bodies were washed three times with PBS and fixed in 5% glutaraldehyde for 24 h. After three times washing with PBS, catheters were dried in a 37 °C incubator and sputter-coated with 10 nm of gold. All samples were observed and photographed on SEM (Phenom XL, Holland)^7^.

### Statistical analysis

All experiments were performed in triplicate and repeated three times. Data were represented as mean ± SD from three independent experiments and analyzed by Graph-Pad Prism software version 8 (San Diego, CA, United States). Statistical comparison among groups was performed by One-Way ANOVA and Student t-test. Differences in the degree of biofilm formation were examined by the Friedman test, followed by the Wilcoxon signed ranks test. A *p-value* < 0.05 was considered as significant.

### Supplementary Information

Supplementary Information.

## Data Availability

All data generated or analyzed during this study are included in this published article.

## Abbreviations

MRSA: Methicillin-Resistant *Staphylococcus aureus*
AMPs: Antimicrobial peptides
mKLK: Modified KLK
MIC: Minimum inhibitory concentration
MBC: Minimal bactericidal concentration
qRT-PCR: Quantitative real-time PCR
TB: Tuberculosis
XDR: Extensively drug-resistant
TDR: Totally drug-resistant
MIC: Minimum inhibitory concentration
MBEC: Minimum biofilm eradication concentration
TSS: Toxic shock syndrome
SCCmec: Staphylococcal cassette chromosome mec
KLK: KLKLLLLLKLK
mKLK: Modified KLK
CLSI: Clinical and Laboratory Standards Institute
TSB: Tryptone Soy Broth
PBS: Phosphate-buffered saline
SD: Standard deviation
