# Supplementary material for: Design of a novel analogue peptide with potent antibiofilm activities against Staphylococcus aureus based upon a sapecin B-derived peptide
Source: Sci Rep. 2024 Jan 26;14:2256. doi: 10.1038/s41598-024-52721-0 (PMC10817945; doi:10.1038/s41598-024-52721-0)

Supplementary Materials

Design of a Novel Analogue Peptide with Potent Antibiofilm Activities against *Staphylococcus aureus* Based Upon a Sapecin B-derived Peptide

Running title: A Novel Analogue Peptide with Potent Antibiofilm Activity

Nasim Akhash[1, 2], Ahmad Farajzadeh Sheikh[1, 2], Zahra Farshadzadeh[1, 2*]

[1]Infectious and Tropical Diseases Research Center, Health Research Institute, Ahvaz Jundishapur University of Medical Sciences, Ahvaz, Iran

[2]Department of Microbiology, Faculty of Medicine, Ahvaz Jundishapur University of Medical Sciences, Ahvaz, Iran

*Corresponding author:
Zahra Farshadzadeh
Department of Microbiology, Faculty of Medicine, Ahvaz Jundishapur University of Medical Sciences, Ahvaz, Iran
Tel: +989161110132
Email: Zahra.farshadzadeh@gmail.com

Supplementary table S1. The clinical source, resistance pattern and molecular characteristics of *S. aureus* isolates

Isolates	Clinical Source	SCC mec		Antimicrobial susceptibility testing[1]												
		mecA	mecC	AMC	AMP	FEP	FOX	GEN	C	E	CIP	TE	LZD	RP	SXT	VAN
MRSA-1	Wound	+[3]	-	R[2]	R	R	R	S	S	S	S	R	S	S	S	S
MRSA-2	Wound	+	-	R	R	R	R	S	S	S	R	R	S	S	S	S
MRSA-3	Wound	+	-	R	R	R	R	S	S	S	S	R	S	S	R	S
MSSA-1	Wound	-	-	S[3]	S	S	S	S	S	S	S	R	S	S	S	S
MSSA-2	Wound	-	-	S	S	S	S	S	S	S	S	R	S	S	S	S

[1]AMC: Amoxicillin/clavulanic acid, AMP: Ampicilln, FEP: Cefepime, FOX: Cefoxicitin, CAZ: Ceftazidime, AMK: Amikacin,

GEN: Gentamicin, C: Chloramphenicol, E: Erythromycin, CIP: Ciprofloxacin, TE: Tetracycline, LZD: Linezolide, RP: Rifampicin,

SXT: Trimethoprim/sulphamethoxazole, VAN: Vancomycin.

[2]R: Resistant, S: Susceptible,

[3]+: Positive, -: Negative

Supplementary table S2. The MIC values of vancomycin against tested *S. aureus* strains

Strains	MIC (µg/ml)	MBC (µg/ml)
ATCC 25923	0.25	0.25
MRSA-1	0.25	0.5
MRSA-2	0.5	0.5
MRSA-3	0.5	0.5
MSSA-1	0.25	0.25
MSSA-2	0.25	0.25

Supplementary table S3: Absorbance of blood sample at 570 nm treated whith KLK, mKLK and LL-37 peptides was measured by spectrophotometry and the degree of hemolysis was calculated. Experiment was repeated three times for each concentration. Optimal density (OD) in repeats 1 to 3 are labelled as OD_1, OD_2 and OD_3.

**KLK peptide**					
Peptide concentration (μM)	OD_1	OD_2	OD_3	Mean± SD	Hemolysis (%)
1.56	0.222	0.224	0.22	0.22± 0.002	10.4
3.12	0.345	0.348	0.346	0.34± 0.001	18.3
6.25	0.482	0.491	0.485	0.48± 0.004	27.1
12.5	0.597	0.604	0.601	0.60± 0.003	34.2
25	0.735	0.741	0.738	0.73± 0.003	42.8
50	1.195	1.188	1.191	1.19± 0.003	71.5
75	1.31	1.361	1.339	1.33± 0.02	80.1
100	1.44	1.47	1.48	1.46± 0.02	88.3
150	1.641	1.637	1.622	1.63± 0.01	92.2
200	1.511	1.521	1.519	1.51± 0.005	95.4
Triton X-100	1.61	1.57	1.59	1.59± 0.02	100
PBS	0.055	0.063	0.051	0.056± 0.006	0
**mKLK peptide**					
1.56	0.058	0.066	0.061	0.06±0.004	0
3.12	0.062	0.065	0.058	0.06± 0.003	0.3
6.25	0.088	0.085	0.081	0.08± 0.003	1.8
12.5	0.204	0.199	0.206	0.20± 0.003	9.3
25	0.303	0.296	0.306	0.30± 0.005	15.4
50	0.635	0.641	0.628	0.63± 0.006	36.2
75	0.816	0.823	0.827	0.82± 0.005	48.2
100	0.977	0.984	0.983	0.98± 0.003	58.2
150	1.045	1.052	1.048	1.04± 0.003	62.4
200	1.125	1.124	1.122	1.12± 0.001	67.1
Triton X-100	1.61	1.57	1.59	1.59± 0.02	100
PBS	0.055	0.063	0.051	0.056± 0.006	0
**LL-37 peptide**					
1.56	0.055	0.064	0.049	0.06± 0.007	0
3.12	0.056	0.058	0.057	0.06± 0.001	0.1
6.25	0.078	0.076	0.079	0.07± 0.001	1.3
12.5	0.132	0.123	0.131	0.12± 0.004	4.5
25	0.227	0.224	0.229	0.22± 0.002	10.6
50	0.378	0.377	0.374	0.37± 0.002	20.1
75	0.512	0.515	0.517	0.52± 0.002	28.7
100	0.585	0.592	0.591	0.59± 0.003	33.5
150	0.838	0.841	0.836	0.83± 0.002	49.1
200	1.053	1.049	1.055	1.0± 0.003	62.6
Triton X-100	1.61	1.57	1.59	1.59± 0.02	100
PBS	0.055	0.063	0.051	0.05± 0.006	0

Supplementary table S4: Inhibition of bacterial attachment at different concentrations of KLK and mKLK peptides was assessed by measurement of crystal violet absorbance at 570 nm. Each concentration was assessed three times. Optimal density (OD) in repeats 1 to 3 are labelled as OD_1, OD_2 and OD_3.

Isolates		Control	1/2MIC		1/4MIC		1/8MIC	
			KLK	mKLK	KLK	mKLK	KLK	mKLK
ATCC25923	OD1	2.148	2.001	1.502	2	1.785	2.05	2.001
	OD2	2.127	1.89	1.462	1.946	1.81	2.09	1.989
	OD3	2.133	1.823	1.47	1.949	1.774	1.95	1.867
	Mean±SD	2.136±0.01	1.904±0.08	1.478±0/02	1.965±0.03	1.789±0.01	2.03±0.07	1.952±0.07
MRSA-1	OD1	1.849	1.546	1.4	1.721	1.671	1.7	1.699
	OD2	1.847	1.506	1.334	1.718	1.619	2	1.733
	OD3	1.889	1.472	1.35	1.698	1.509	1.701	1.739
	Mean±SD	1.861±0.02	1.508±0.03	1.361±0.03	1.712±0.01	1.599±0.08	1.8±0.17	1.723±0.02
MRSA-2	OD1	2.314	2.103	1.493	2.141	1.611	2.223	2.06
	OD2	2.303	2.094	1.485	2.156	1.823	2.221	1.86
	OD3	2.316	2.051	1.51	2.143	2.001	2.15	1.799
	Mean±SD	2.311±0.005	2.082±2.02	1.496±0.01	2.146±0.008	1.811±0.19	2.198±0.04	1.906±0.13
MRSA-3	OD1	1.422	1.118	0.943	1.386	1.103	1.501	1.242
	OD2	1.505	0.99	1.019	1.362	1.14	1.407	1.115
	OD3	1.547	0.987	0.87	1.36	0.992	1.378	1
	Mean±SD	1.491±0.06	1.031±0.07	0.944±0.07	1.369±0.01	1.078±0.07	1.428±0.06	1.119±0.12
MSSA-1	OD1	2.033	1.889	1.23	1.999	1.429	2.006	1.319
	OD2	2.09	1.893	1.307	1.894	1.398	1.988	2.007
	OD3	2.2	1.89	1.15	1.882	1.431	2.026	1.451
	Mean±SD	2.107±0.08	1.89±0.002	1.229±0.07	1.925±0.06	1.419±0.01	2.006±0.01	1.592±0.36
MSSA-2	OD1	2.076	1.81	1.094	1.364	1.364	2.018	1.099
	OD2	2.066	1.831	1.277	1.501	1.501	1.983	2.03
	OD3	2.043	1.771	1.226	1.413	1.413	1.874	1.6
	Mean±SD	2.061±0.01	1.804±0.03	1.199±0.09	1.426±0.06	1.426±0.06	1.958±0.07	1.576±0.46

Supplementary table S5: Inhibition of biofilm formation at different concentrations of KLK and mKLK peptides was assessed by measurement of crystal violet absorbance at 570 nm. Each concentration was assessed three times. Optimal density (OD) in repeats 1 to 3 are labelled as OD_1, OD_2 and OD_3.

Isolates		Control	1/2MIC		1/4MIC		1/8MIC	
			KLK	mKLK	KLK	mKLK	KLK	mKLK
ATCC25923	OD1	2.31	1.653	0.221	2.071	0.227	2.113	0.694
	OD2	2.298	1.649	0.218	2.112	0.221	2.104	0.657
	OD3	2.3	1.662	0.182	2.024	0.324	2.124	0.684
	Mean±SD	2.302±0.006	1.654±0.006	0.207±0.02	2.069±0.04	0.257±0.05	2.113±0.01	0.678±0.01
MRSA-1	OD1	2.103	1.587	0.487	1.928	0.561	2.001	1.023
	OD2	2.122	1.611	0.489	1.919	0.58	1.94	0.9
	OD3	2.122	1.62	0.484	1.926	0.577	1.9	0.886
	Mean±SD	2.115±0.01	1.606±0.01	0.489±0.007	1.924±0.004	0.572±0.01	1.947±0.05	0.936±0.07
MRSA-2	OD1	2.37	1.86	0.434	2.142	0.599	2.187	0.862
	OD2	2.393	1.869	0.449	2.109	0.603	2.19	1.007
	OD3	2.3	1.855	0.45	2.133	0.67	2.186	0.902
	Mean±SD	2.354±0.04	1.861±0.007	0.444±0.008	2.128±0.01	0.624±0.03	2.187±0.002	0.923±0.07
MRSA-3	OD1	1.516	0.183	1.398	1.742	0.301	1.391	0.512
	OD2	1.563	0.189	1.39	1.739	0.318	1.481	0.524
	OD3	1.545	0.245	1.427	1.738	0.347	1.451	0.523
	Mean ±SD	1.541±0.02	0.204±0.03	1.405±0.01	1.739±0.002	0.322±0.02	1.441±0.04	0.519±0.006
MSSA-1	OD1	2.2	1.637	0.197	2.098	0.415	2.13	0.596
	OD2	2.169	1.571	0.241	1.899	0.406	1.86	0.632
	OD3	2.202	1.62	0.271	1.918	0.4	2.031	0.638
	Mean±SD	2.190±0.01	1.609±0.03	0.236±0.03	1.971±0.10	0.407±0.007	2.007±0.13	0.622±0.02
MSSA-2	OD1	1.823	1.28	0.197	1.643	0.217	1.69	0.497
	OD2	1.777	1.263	0.181	1.62	0.212	1.612	0.584
	OD3	1.808	1.241	0.173	1.62	0.248	1.599	0.593
	Mean±SD	1.802±0.02	1.261±0.01	0.183±0.01	1.627±0.01	0.225±0.01	1.633±0.04	0.558±0.05

Supplementary table S6: Dispersal activity of KLK and mKLK peptides at different concentrations (1 MIC, 2 MIC and 4 MIC) was assessed by measurement of crystal violet absorbance at 570 nm. Each concentration was assessed three times. Optimal density (OD) in repeats 1to 3 are labelled as OD_1, OD_2 and OD_3.

Isolates		Control	1MIC		2MIC		4MIC	
			KLK	mKLK	KLK	mKLK	KLK	mKLK
ATCC25923	OD1	2.822	2.459	1.294	2.344	0.944	2.01	0.36
	OD2	2.75	2.444	1.276	2.303	0.637	2.012	0.359
	OD3	2.799	2.451	1.262	2.321	0.791	2.031	0.333
	Mean±SD	2.79±0.03	2.451±0.007	1.277±0.01	2.322±0.02	0.790±0.15	2.017±0.01	0.35±0.01
MRSA-1	OD1	1.903	1.75	1.297	1.725	1.01	1.439	0.832
	OD2	1.9	1.701	1.26	1.599	0.881	1.418	0.661
	OD3	1.907	1.636	1.258	1.623	0.888	1.422	0.27
	Mean±SD	1.903±0.003	1.695±0.05	1.271±0.02	1.649±0.06	0.926±0.07	1.426±0.01	0.587±0.28
MRSA-2	OD1	2.03	1.812	1.402	1.722	1.031	1.65	0.576
	OD2	2	1.803	1.342	1.7	0.901	1.649	0.499
	OD3	2.013	1.799	1.305	1.71	0.857	1.632	0.662
	Mean±SD	2.014±0.01	1.804±0.006	1.349±0.04	1.71±0.01	0.929±0.09	1.643±0.01	0.579±0.08
MRSA-3	OD1	1.109	1.1	0.521	1.02	0.439	0.97	0.286
	OD2	1.596	1.116	0.552	1.08	0.424	0.968	0.3
	OD3	1.06	1.149	0.819	1.2	0.637	1.05	0.303
	Mean±SD	1.255±0.29	0.121±0.02	0.637±0.16	1.1±0.09	0.5±0.11	0.996±0.04	0.296±0.009
MSSA-1	OD1	1.913	1.599	1.091	1.503	0.673	1.367	0.377
	OD2	1.708	1.573	1.142	1.466	0.651	1.334	0.4
	OD3	1.74	1.597	0.957	1.48	0.701	1.344	0.357
	Mean±SD	1.787±0.1	1.589±0.01	1.063±0.09	1.483±0.01	0.675±0.02	1.348±0.01	0.378±0.02
MSSA-2	OD1	2.123	1.999	1	1.79	0.873	1.522	0.207
	OD2	2.115	1.79	0.971	1.679	0.501	1.52	0.218
	OD3	2.111	1.787	1.007	1.672	0.539	1.52	0.197
	Mean±SD	2.116±0.006	1.858±0.12	0.992±0.01	1.713±0.06	0.637±0.20	1.52±0.001	0.207±0.01

Supplementary figure 1: Crystal violet assay to investigate the dispersal activity of KLK (a) and mKLK (b) peptides

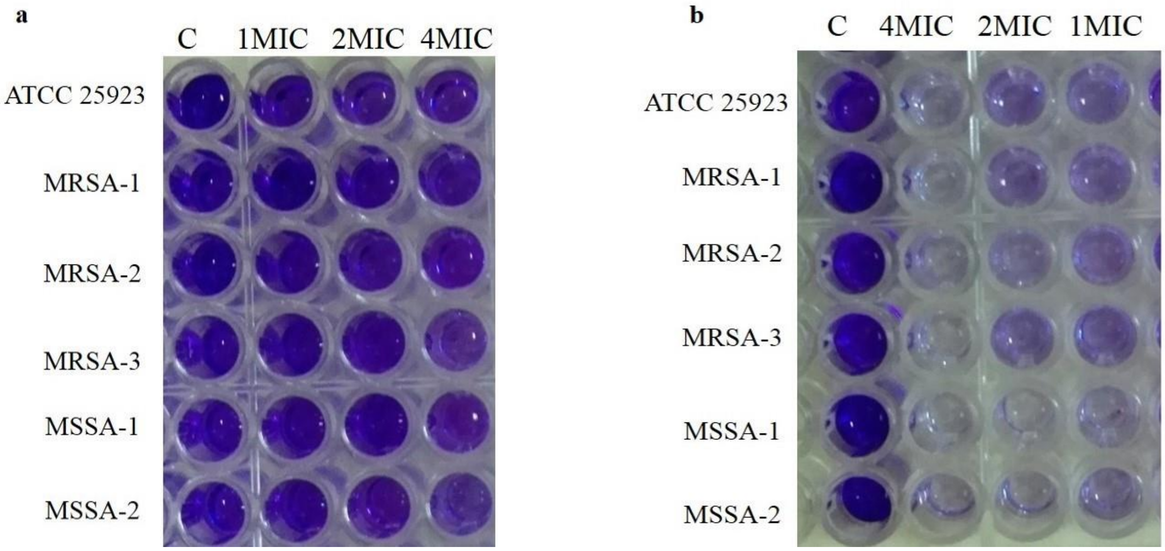

Supplement: Supplementary file 1 — Supplementary Information. [file 41598_2024_52721_MOESM1_ESM.pdf]
